# Perspectives of religious beliefs and family planning by religious leaders and young women: results from a qualitative study of Bobo-Dioulasso and Ouagadougou in Burkina Faso

**DOI:** 10.1186/s12889-025-26160-z

**Published:** 2026-01-08

**Authors:** Amelia Maytan-Joneydi, Kindo Boukary, Fiacre Bazié, Fatimata M. Traoré, Yentema Onadja, Georges Guiella, Ilene S. Speizer

**Affiliations:** 1 Carolina Population Center, University of North Carolina at Chapel Hill, Chapel Hill, NC USA; 2 Institut Supérieur des Sciences de la Population (ISSP), Université Joseph Ki-Zerbo, Ouagadougou, Burkina Faso; 3 Department of Maternal and Child Health and Carolina Population Center, University of North Carolina at Chapel Hill, Chapel Hill, NC USA

**Keywords:** Family planning, Adolescents, Youth, Religion, Religious leaders, Burkina faso

## Abstract

**Background:**

Burkina Faso is religiously diverse compared to its neighbors. Religion and religious leaders are often perceived as being against family planning (FP) use. That said, the literature on different religions and religious leaders’ position on FP does not demonstrate a uniform stance and there is a dearth of perspectives related to use among young people.

**Methods:**

Qualitative data for this study were collected in July 2022 in Ouagadougou and Bobo-Dioulasso, Burkina Faso. Focus group discussions (FGD) were conducted with Catholic, Protestant, and Muslim young women who were aged 18–24 and were current users of FP. In-depth interviews (IDI) were conducted with pro-FP Catholic, Protestant, and Muslim religious leaders. The FGD and IDI data were translated and transcribed into French, thematically coded, and qualitatively analyzed.

**Results:**

Young women of all faiths generally perceived their religions were against FP use for anyone, and especially for unmarried women. There was some openness to FP use by married women for birth spacing, especially with the consent of the husband. When young women were asked about their reasons for using FP despite their perception of their religion’s position against it, married young women said they used FP due to financial and practical difficulties inherent to modern day life, and to promote a more harmonious family life. Unmarried young women used FP to avoid social stigma and to pursue their own studies. IDI with pro-FP religious leaders of all faiths demonstrated their support of FP; they spoke of its benefits. While religious leaders did not say their religion prohibits FP use, they expressed some conditions on their support of FP such as marital status, purpose of FP use, and type of method used.

**Conclusions:**

This study shows that while young Christian and Muslim women largely perceive their religion to be against FP use, other factors are considered in their decision to use. While religious leaders remain influential, for young women, societal level pressures are more pressing. Religious leaders and other stakeholders should consider the current needs of young people and their own evolving beliefs around FP in their messages to their communities.

**Supplementary Information:**

The online version contains supplementary material available at 10.1186/s12889-025-26160-z.

## Background

Burkina Faso is a Sahelian country in the West African region of sub-Saharan Africa. Burkina Faso had a population of 22.9 million people, and a total fertility rate of 4.4 in 2022 [1]. In 2021, 27.9% of all women ages 15–49 in Burkina Faso used a modern method of contraception and among young women, 11.6% of women aged 15–19 and 32.3% of women aged 20–24 used a modern method of contraception [2]. Family planning (FP) use provides many benefits to young women and adolescents and has been shown to lead to reductions in maternal deaths and morbidity, and improved infant and child health and survival [3]. Starting with FP2020 and now FP2030, Burkina Faso has made commitments to increase contraceptive use in the country through increased advocacy, budget al.locations, and improved access to contraceptives and sexual and reproductive health services [4]. Since 2012, the rate of modern contraceptive use among all women has increased by 12% points, [5] still unmet need for FP, that is, the percentage of women who want to delay or stop childbearing and are not using FP, remains at 13.1% of all women with an unmet need for spacing and 3.3% with an unmet need for limiting [6]. Among younger women ages 15–19, 6% had an unmet need for spacing; this value is 17.5% among their peers ages 20-24. [6]

Burkina Faso is religiously more diverse than many of its neighbors with about 63% of the population being Muslim, 25% Catholic, and 8% being Protestant [2]. In many studies from sub-Saharan Africa, South Asia, and Europe, both in Christian and Muslim contexts, religion and religious leaders have been reported to be against FP use or to be a reason for non-use or unmet need for FP. [7–14] The social-ecological model (SEM) developed by Urie Bronfenbrenner and further developed for health promotion by McLeroy et al. posits that there are multiple layers of influence on an individual’s behavior which include individual/intrapersonal, interpersonal, institutional, community, and policy factors [15, 16]. Religion and religious leaders exert influence at the individual level through personal beliefs as well as the community level through social pressure. At the individual level, religion can influence women’s contraceptive use through their own personal and religious beliefs around fertility, and knowledge of FP. A qualitative study using participant observation and in-depth interviews with male and female inhabitants of a village in Pakistan affirmed the commonly held belief that family planning is contrary to Islam’s teachings [7]. Another qualitative study found that men and women in rural Uganda believe that children are a gift from God and that both Christianity and Islam view FP use as contrary to the will of God [9]. Furthermore, religion and religious leaders can affect the interpersonal and community levels of influence of the SEM through influencing the beliefs of those who surround a woman and have interpersonal relationships with her, and through the community level of influence, by affecting social norms and acceptable behaviors within religious communities. Also in rural Uganda, another qualitative study found that women and men of reproductive age and health workers reported that both Christian and Muslim religious leaders discouraged FP use [13]. Similarly, in a recent study from Burkina Faso, religious leaders were found to disapprove of FP use and to discourage its use [17]. Few studies were found that look at religion and FP use for young and unmarried women. However, in a mixed methods study in a university setting in Zimbabwe, students, in particular those of Pentecostal and Protestant faiths, expressed that religion commonly hindered FP use [12]. In line with SEM but more specific to religious influences on FP use, McQuillan presents three mechanisms through which religion and religious leaders influence fertility and contraceptive use. First, religion and religious leaders express and articulate norms relevant to fertility and contraceptive use acceptability. Second, religious leaders have a platform to communicate these norms and values and encourage compliance with their views. Third, where religion is an important determinant of community engagement, these influences are stronger on members of the community [18]. 

While many studies have shown that religion negatively influences contraceptive use, the literature around religion’s position on FP use does not reveal a uniform stance [11, 19–22]. Pinter et al. (2016) conducted a review of several religions’ views on FP and found diverse positions. The Pinter et al. (2016) review addresses the first mechanism outlined by McQuillan through which religion influences contraceptive use by exploring each religion’s doctrine about fertility and contraception. The review found that within Christianity, Catholicism does not allow for use of FP except for the rhythm method, and Protestantism allows for use of all modern methods for married women. In Islam, the use of modern methods, except for sterilization, was found to be acceptable for married women if the quality of life or health of women or children were compromised [23]. While Catholic doctrine is clear in its position on FP with only abstinence and the rhythm method deemed as acceptable, for Protestantism and Islam, interpretation of religious doctrine and principles plays an important role in influencing perceptions of religious acceptability of FP.^23^ The role that personal interpretation by religious leaders plays in influencing their religious communities’ perceptions of FP use is affirmed by the finding that contraceptive use varies more by congregation rather than by denomination [24]. In an examination of the influence of religion on contraceptive use, religion was not influential on use by denomination but a closer look by congregation found that congregants ever use of FP was strongly related to their religious leader’s beliefs around FP.^24^ Societal influences and socioeconomic factors contribute to FP norms and patterns among religious communities as differences in contraceptive practices have been documented among Muslim populations in Sub-Saharan Africa compared to in Asia [24]. A qualitative study from Tanzania found that among Christian and Muslim men and women, religious teachings were used to advocate both against and in favor of the use of FP.^22^ Both Christian and Muslim participants expressed that using FP is going against God’s plan and preventing children from being born. Others believed that FP is consistent with their religious and moral duties to properly care for their children and live within their means [22]. A qualitative study from two predominantly Muslim counties in Kenya also revealed divergent opinions among religious leaders and women and men in the community in regard to the acceptability of FP use [19]. The literature does not provide clear consensus on religion’s position or religious followers’ position on FP use, particularly among youth and unmarried people.

The rationale behind this study is to understand how religion and religious leaders influence young women’s beliefs and decisions around FP by comparing young women’s perceptions of their religion’s position on FP to religious leaders’ perspectives on FP. The objective of this qualitative study is to explore the perspectives of practicing young Christian (Catholic and Protestant) and Muslim women on their religion’s position on FP use and on their own reasons for use. Alongside this exploration of young women’s perceptions on FP use, this study also examines religious leaders’ stance on FP use– for whom is it acceptable, in which circumstances, which methods are sanctioned, and specifically what is their view on young people using FP.

## Methods

### Study setting

The data presented in this paper were collected from two cities in Burkina Faso: Ouagadougou in Centre Region, and Bobo-Dioulasso in Hauts-Bassins Region. The two study sites were selected as they are the two largest cities in Burkina Faso (Ouagadougou is the capital) with religious diversity and comparatively high levels of adolescent and youth FP use compared to nationally.

### Study design

This qualitative study includes data from 18 in-depth interviews (IDI) conducted with religious leaders and eight focus group discussions (FGD) with young women who were ages 18–24 and current users of FP at the time of the study. IDI were conducted with religious leaders of different faiths– Catholic, Protestant, and Muslim— who had previously participated in activities to promote FP and were considered to be “pro-FP”. Efforts were made to recruit an equal number of religious leaders from each faith and also to recruit female and male religious leaders. FGD were conducted with young women aged 18–24 of different religious backgrounds and marital statuses and who were using a modern method of FP at the time of the study. FGD were stratified by religion and marital status with Catholic and Protestant participants together as Christian.

### Interview guide development and study team

The study team is made up of Burkinabe investigators (KB, FB, FT, YO, GG) and two U.S.-based collaborators (AMJ, ISS). One of the study authors (KB) served as a supervisor during data collection and another (FT) was an interviewer for the study. All six interviewers were from Burkina Faso and familiar with the study sites and local languages. The semi-structured interview guide for religious leaders and the discussion guide for the FGD with young women were developed collaboratively for this study by all team members from the Institut Supérieur des Sciences de la Population (ISSP) in Burkina Faso, and the Carolina Population Center at the University of North Carolina, Chapel Hill (UNC-CPC). All guides were piloted in Ouagadougou, Burkina Faso before the interviewer training began, and then once again during the interviewer training to ensure a logical flow of the guides and the acceptability of the themes discussed. Minor modifications were made to the guides based on feedback from the two rounds of piloting. IDI and FGD guides are provided in the supplemental material and covered perspectives on religion and contraceptive use for young people.

### Study participants and sampling

Prior to the beginning of data collection, ISSP’s study team sent letters introducing the study to leadership at each of the health districts where data collection was taking place. The study team engaged community health workers at each site to get their help in identifying eligible young women ages 18–24 who were current users of FP to participate in the FGD. Study teams then screened identified young women and set a date and time for the FGD based on religion and marital status. To be eligible to participate, the young women had to be active participants in their religious community. For Christians this meant they had to self-report attending church weekly, and for Muslims praying five times a day. Young women were also required to report that they participated in activities within their religious community as well. Table 1 describes the marital status and religions of participants in the FGD. Women who were not eligible to participate in the FGD were given a beverage and thanked for their interest in participating.

Recruitment for IDI with religious leaders was done with the help of the Union des Religieux et Coutumiers du Burkina (URCB). URCB is an organization that has worked with the Ministry of Health to support their work in the domain of sexual and reproductive health. After being introduced to the study, URCB provided the study team with a list of religious leaders they considered to be pro-FP, and the study team reached out to religious leaders individually to explain the objectives of the study and request their participation. Religious leaders that accepted to participate in the study were given an appointment to meet for the IDI at a location of the study participant’s choosing.

### Data collection

Data were collected in July of 2022 by ISSP. ISSP conducted training for the interviewers which included training on research ethics, qualitative research methods, the study objectives, and in-depth understanding of the interview guides. The interviewers had previous experience with qualitative research data collection. Six trained female interviewers divided into two teams (one team for each city) to conduct the IDI with religious leaders. The same six interviewers worked in teams of two (one facilitator and one note taker) to conduct the FGD. All participants provided written informed consent before participating in the study; all IDI and FGD were audio recorded. The IDI and FGD were held in varying locations depending on the needs of the study participants but were all completed in an area that provided privacy to study participants to permit them to be able to speak freely. The IDI and FGD were conducted in French, Dioula, or Mooré depending on the preference of study participants. Interviews with religious leaders lasted between 43 and 85 min, and FGD were between 58 and 85 min long. The FGD were stratified by marital status and religious background, the different FGD are detailed in Table 1 below.

**Table 1:** Description of young women who were current users aged 18–24 included in FGD data collection, Bobo-Dioulasso and Ouagadougou, Burkina Faso

Group Number	Marital Status	Religious Background	City of Residence	Number of Participants
1	Unmarried	Christian	Bobo-Dioulasso	8
2	Married	Christian	Bobo-Dioulasso	9
3	Unmarried	Muslim	Bobo-Dioulasso	8
4	Married	Muslim	Bobo-Dioulasso	11
5	Unmarried	Christian	Ouagadougou	8
6	Married	Christian	Ouagadougou	8
7	Unmarried	Muslim	Ouagadougou	8
8	Married	Muslim	Ouagadougou	8

### Data analysis

Audio recordings of the IDI and FGD were transcribed into French by the interviewers themselves immediately following the IDI or FGD. All transcripts then were uploaded into Dedoose, a collaborative qualitative analysis software, for coding and analysis. Preliminary code books for both IDI and FGD analysis were created based off the corresponding interview and discussion guides. The codebooks were then reviewed by team members for input and then uploaded into Dedoose for coding. An initial transcript was coded by all members of the research team and then the coding was reviewed during group meetings to examine discrepancies and clarify codes. This process was repeated with a second transcript for each tool to attain agreement among coders and to add any necessary additional codes. After the review process, the transcripts were divided up between the coders to be separately coded. This coding process was the same for both the IDI and FGD transcripts. Following coding, thematic analysis was then completed. In addition, memos were written summarizing the themes discussed in the IDI with religious leaders. Different matrices were created for the IDI and FGD data and themes and were extensively discussed in team meetings. Results were examined by religious affiliation, age, and marital status (for young women). The quotes presented in this paper were translated from French into English and the translation was reviewed and approved by the Burkinabé study team.

### Ethical approval

Ethical approval for all consent forms, IDI interview guides, FGD discussion guides, and study protocols was given by the Comité d’Ethique pour la Recherche en Santé in Burkina Faso (#2022-06-122) on 13 June 2022 and by the Institutional Review Board at UNC on 23 May 2022 (#22-1125).

## Results

### Participant characteristics

This study had two groups of participants: religious leaders who participated in IDI and young women users who participated in FGD. Religious leaders interviewed ranged in age from 36 to 61 years. The majority of leaders interviewed were men, with women religious leaders making up a third of the sample. There was an equal number of Muslim and Protestant religious leaders interviewed but only two Catholic leaders were interviewed. Catholic leaders were more reticent to participate in this study than other religious leaders. The characteristics of the religious leaders across the two study cities are presented in Table 2.

**Table 2:** Characteristics of religious leaders interviewed in Bobo-Dioulasso and Ouagadougou, Burkina Faso

Characteristics of religious leaders	Bobo-Dioulasso	Ouagadougou	Total
Age			
30–39	1	1	2
40–49	1	3	4
50–59	6	3	9
60–69	2	1	3
Sex			
Female	4	2	6
Male	6	6	12
Religion			
Catholic	2	0	2
Protestant	4	4	8
Muslim	4	4	8

Eight FGD were conducted with young female current users of a modern FP method aged 18–24. The median age was 23 years, and the mean was 22 years with a standard deviation of 1.9 years. By design, approximately half of participants were Muslim and the other half was Christian in both cities, with more Catholic than Protestant participants. Likewise, around half of the young women were married. Over two thirds of the young women had one or more children. All participants were current users of a FP method, with the implant as the most commonly used method among participants in Bobo-Dioulasso and the condom as the most commonly used method in Ouagadougou. The young women included from Bobo-Dioulasso were less educated than those interviewed from Ouagadougou as can be seen in Table 3. More details about the participant characteristics are presented in Table 3.

**Table 3:** Characteristics of FGD participants in Bobo-Dioulasso and Ouagadougou, Burkina Faso

Characteristic of FGD participants	Bobo-Dioulasso	Ouagadougou	Total
Age			
18–20	8	7	15
21–22	9	6	15
23–24	19	19	38
Parity			
0	11	12	23
1	19	9	28
2	6	11	17
Current method			
Injectable	11	8	19
Implant	15	9	24
Pill	7	4	11
Condom	1	10	11
IUD	2	1	3
Level of education			
None	18	8	26
Primary	5	5	10
Post-primary	9	4	13
Secondary	3	13	16
University	1	2	3

### Young women’s perceptions of their religion’s position on FP use

In all of the FGD, the main sentiment expressed among the young women from all religious backgrounds was that their religious communities are not in favor of FP use for married as well as unmarried women. The young women shared that their religion says that “FP is not a good thing,” that it is “a sin,” and that it is “forbidden.” The reasoning behind this position, regardless of religious background, was that children are a “gift from God” and trying to influence motherhood would be a form of disobedience to God. When a woman gets married, she is expected to begin having children; using FP would reduce the number of children that she is predestined to have and would thereby favor her own will over God’s will. For example, the following two quotes from a FGD with young married Muslim women and from a FGD with young unmarried Christian women:

*“In the Muslim religion it is said that family planning is forbidden…Muslim leaders have said to not use family planning*,* that it allows a woman to limit births and it allows you to harm the children that God intended for you to give birth to*,* which is not good in Islam.” -* Married Muslim young woman from Ouagadougou.

*“So! The ban in assemblies is as if to say*,* if God allows you to give birth*,* you must give birth. If you take contraception when God had only one child reserved for you*,* after having had this child you take contraception and then you can no longer have children and you complain to God…At church they are not for its use because it is God who gave you procreation so regardless of when or how you must simply procreate.” -* Unmarried Christian young woman from Ouagadougou.

The young women’s views on their religion’s position on FP were based on a complex combination of factors such as their and their social network’s interpretation of religious texts, preaching by religious leaders, and conversations within their community. These factors shape their perception of FP, and in turn influence their decisions about motherhood and birth spacing. Passages of religious scripture play a role in informing women’s understanding of FP; women cited texts from scripture such as “Multiply yourselves and replenish the earth” as meaning that women should have as many children as possible without constraints. This literal understanding of these sacred texts has an impact on their attitude and perception of birth spacing or limitation. A young Catholic woman spoke of priests who, during Eucharistic celebrations, discussed birth spacing and proclaimed that FP was forbidden. These religious speeches by key religious figures shape young women’s understanding of FP and what their religious community feels about FP.

In addition to the perception that FP use is prohibited for all women, there were very clear views that FP use by unmarried women was unacceptable among participants across the religions. This stance stems from the belief that premarital sexual activity is not allowed and accepting young unmarried women’s use of FP would be tantamount to encouraging extra-marital sexual relations which is contrary to religious teachings. An unmarried protestant woman explained that religion tells unmarried women not to use FP because the only reason to use FP would be if they were sexually active. This same reasoning was shared by both married and unmarried women across all religions.

*“The obstacle is that in the gospel it is said that everyone must save themselves until marriage without knowing a man. And for this you do not need to use contraception since you must save yourself and if you do [use contraception] it means that you want to sin. This is also the blockage.” -* Unmarried Catholic young woman from Ouagadougou.

#### Nuances of religions’ positions on FP use

As discussed above, while most young women in the FGD felt that their religion opposed FP use, regardless of marital status, some participants from all religious groups expressed that their religious community allows for FP use specifically for married women. One FGD of married Christian young women had a discussion of how married women can use FP, especially when it is agreed upon with their husband. Likewise, in a Muslim FGD with married participants, several young women expressed that once a woman is married, she is accountable to her husband and if he consents to her use of FP then it is religiously allowed.

*“In our religion if you are married you can use*,* but if you are not married and you use*,* the consequences will be enormous. If you are married*,* it is between you and your husband*,* it is you who see together.” -* Married Christian young woman from Bobo-Dioulasso.

*" At the mosque they say that after God your husband comes next. He is your second god. If he gives you permission*,* the mosque has nothing more to say. Your husband prays to God*,* you pray to your husband*,* if he has accepted there is no problem.” -* Married Muslim young woman from Bobo-Dioulasso.

In focus group discussions, both Christian and Muslim young women spoke of birth spacing for the health and well-being of mothers and children as an acceptable reason for FP use for married women. The young women expressed that having children closely spaced together is not a desirable outcome for women themselves, their husbands, or religious leaders. For example, this woman talks about acceptable circumstances for FP use in a Christian FGD of unmarried women.

*“At our church*,* we talk about that. You can have children*,* but when it’s close together*,* the children themselves won’t be comfortable*,* you won’t be comfortable either. Even no pastor will encourage you in this direction; they talk to us about that.” -* Unmarried Christian young woman from Bobo-Dioulasso.

And this group of married Muslim women also talked about FP use being acceptable for purposes of birth spacing.

*“When I gave birth*,* it was my husband who told me to use FP*,* he is Muslim and practicing. Where I live*,* I don’t see Muslim leaders who say it’s not good. Moreover*,* according to some Muslim practitioners*,* FP is good*,* you manage to space your births…otherwise if the children are very close together it is not good and above all how are you going to manage to take care of them…”-* Married Muslim young woman from Ouagadougou.

### Religious leaders’ positions on FP use and the basis of their positions on FP

The religious leaders interviewed shared a different perspective on the acceptability of FP than those shared by the young women who participated in the FGD. Given the selection criteria for religious leaders, the majority of leaders believed that the concept of FP is accepted by their religion and all leaders spoke of various benefits of FP. Protestant and Muslim leaders stated that their religion is not against FP. Several leaders, when explaining their own position in support of FP, prefaced it with saying that FP is allowed within their religion.

*“Ok*,* well*,* family planning is a good thing. I would say a very good thing in my opinion and also from the point of view of the religion that we defend*,* that we believe.” -* Christian religious leader from Bobo-Dioulasso (man).

*“FP is a very good thing. Islam does not forbid FP.” -* Muslim religious leader from Bobo-Dioulasso (woman).

Pro-FP religious leaders interviewed from all three religious backgrounds voiced approval and support of FP for the purpose of planning and spacing births. Birth spacing was praised for allowing women to rest and recuperate between births, leading to better health outcomes for children, facilitating increased education, professional, and financial fulfilment for women, leading to children receiving better scholastic, moral, and religious education, contributing to a more harmonious family life, and easing financial burdens on families. Both Christian and Muslim leaders spoke of the difficulties faced by families when they do not space their children. One female Christian leader explained in detail how families can avoid a lot of worries by spacing their children. She described how financial resources are limited in the present day and how any health problem can lead to financial strain within a household. She continued to say that women now need to supplement their husband’s salaries with their own business which is hard to do with several small children close in age. These sentiments were shared by other religious leaders:

*“Anyway*,* spacing out children is good for the health of the woman and the best for the family; for the health of the children.” -* Muslim religious leader from Bobo-Dioulasso (woman).

*“It is even very beneficial*,* because the woman flourishes by doing FP; spacing out births allows children to grow better*,* parents are not stressed*,* children are comfortable. Otherwise if there is a child in the stomach*,* one on the back and another in the arms it becomes difficult to take care of them all at once. FP is beneficial for all of us.” -* Catholic religious leader from Bobo-Dioulasso (man).

#### Nuances of FP acceptability– for whom, in which situations, and using which methods

##### FP use for married women; abstinence for unmarried

Across all religions, acceptance and support of FP was expressed by religious leaders in terms of planning one’s family and was limited to married women. Abstinence is what all religious leaders expressed was the religious expectation for young unmarried people to practice. Sexual activity before marriage is strongly opposed by female and male, Muslim, and Christian religious leaders. A Muslim leader stated that young unmarried people are excluded from the conversation on FP and that their only choice is to practice abstinence. Another Christian leader expressed a similar sentiment:

*“What we teach a lot more to young people in evangelical churches is abstinence. So*,* we don’t advocate*,* we don’t ask young people to plan*,* no. For young people*,* we teach them abstinence because sexuality is reserved strictly within the framework of marriage*,* that’s it.” -* Christian religious leader from Bobo-Dioulasso (man).

##### FP use for birth spacing

In their discussion of FP use for birth spacing, some Muslim leaders would contrast their statement of support for FP with further clarification that use of FP to limit births was not acceptable. The differentiation between birth spacing and limiting births was nuanced. Using FP to space births was described as taking the desired time needed to recover from a previous birth, for the youngest child to grow older, or for finances to be stable before having another child. Limiting births was described as a person or a couple deciding that they want to have a specific number of children or deciding that they do not want any more children. Nuance exists between planning a family and limiting childbearing and the two are considered differently as is expressed by this Muslim religious leader below.

*“Limiting births is prohibited in Islam; it is FP that is authorized. Often people were confused; but we managed to resolve this confusion. FP is about responsible procreation whereas limiting is to say that we want a child and that is enough. However*,* Islam does not completely agree with this. So*,* have children*,* whenever you want; have children if we have the means*,* if we can feed two children*,* we do it*,* that’s three children; depending on our means*,* we carry out responsible procreation. -* Muslim religious leader in Bobo-Dioulasso (man).

Whereas Muslim leaders spoke against use of FP to limit the number of children, Christian leaders did not mention this topic. A couple of Christian leaders discussed the importance of couples talking about the number of children they want and can provide for and then using FP to achieve their reproductive goals.

##### Acceptable FP methods

When discussing which specific FP methods were acceptable, religious leaders demonstrated different levels of familiarity and knowledge of FP methods. Some leaders had experience with use personally, others had backgrounds in health or had participated in FP trainings and were able to name multiple methods and how they work, while a small group spoke of FP more generally without much detail. Both Christian and Muslim religious leaders stated that natural methods, barrier methods and hormonal methods that prevent ovulation are acceptable. However, leaders of all religious backgrounds expressed that any methods that in the words of a leader from Ouagadougou, “touch human life,” and methods that act post fertilization are not allowed.

The two Catholic leaders interviewed expressed approval of a more limited list of FP methods. While being supportive of FP, they stated that the only methods acceptable to the Church were abstinence, lactational amenorrhea method (LAM), and the rhythm method. However, when discussing their personal views on FP, one Catholic leader was pro-FP, having used it for his own family and promoting it among youth. The other Catholic leader described FP as “a necessary evil” in the current state of the world with what he estimates as 70% of young people engaging in sexual activity before marriage.

Several Muslim leaders said that permanent or irreversible FP methods (sterilization) were not acceptable except in the case where a woman’s health and life are at risk. The most commonly mentioned acceptable methods among Muslim leaders were LAM and withdrawal (coitus interruptus) which leaders said are methods mentioned in the Quran and in other religious texts. The standard days method and condoms were also mentioned as being acceptable.

##### Young people and FP

Religious leaders were asked about their positions on FP use by young people. Almost all the religious leaders, while supportive of FP, were adamant that FP use was appropriate for married young people in the context of planning their families and that abstinence is the “method” to be used by young unmarried people until marriage. Several religious leaders expressed discomfort even discussing FP use for unmarried women as sexual activity outside of marriage is not acceptable religiously. Religious leaders talked about how even the term “family planning” indicates that its use is reserved for within a family which in their opinion begins with marriage.

*“For me*,* even when you’re not married*,* we don’t even talk about family planning because we’re talking about family. To be in a family*,* you have to get married; if you’re not married*,* we can’t talk about family. FP concerns the family. If you are single*,* you are not concerned. For you it’s abstinence. But when you’re in your home with your husband*,* it’s up to you*,* what you want to do. It’s up to you to discuss and exchange ideas with each other to plan your births.” -* Muslim religious leader from Bobo-Dioulasso (woman).

*“Us*,* we say that this will be done first within the framework of marriage. If there is no marriage there is no place to talk about family planning. Since the family is first formed*,* it is within the family that we plan.” -* Christian religious leader from Ouagadougou (man).

While almost all the pro-FP religious leaders interviewed clearly spoke against FP use among unmarried young people, differences in opinions about sharing information about FP with unmarried young people were expressed. Most of the leaders stated that they believe that unmarried young people should have access to information. The type of information they believe young people should have access to varied and included information about FP methods, sexuality, marriage and family life, sexual and reproductive health, or FP services. A few leaders said they felt that young people should be informed about FP because premarital sex is frequent now or because they may otherwise get information “from the street” or other less desirable sources. Some leaders felt that unmarried young people should know about FP methods in preparation for marriage, whereas others felt that until they were married or imminently going to be married, young people do not need to know about FP methods specifically.

*“For me*,* we need to raise awareness among these young girls about FP*,* we can give them all the information on contraception*,* and we promote abstinence for these young girls until marriage. Once they are married they have a sex life now they come to planning services to use if they want.” -* Muslim religious leader from Ouagadougou (woman).

Other leaders across the religions stated that unmarried young people only need information about abstinence and expressed concern that giving them information about FP methods would only inspire curiosity and lead to debauchery.

*“It is instead better to give them advice on how to stay chaste until marriage. Why talk about FP to a single girl*,* if the person knows that she can have sex without getting pregnant*,* she will go do it now. She may be afraid to do it under the pretext that she is going to get pregnant*,* but if we give her a method*,* she is no longer afraid.” -* Muslim religious leader from Ouagadougou (woman).

#### Basis of religious leaders’ position on FP

While explaining their positions on FP, none of the leaders cited religious texts that specifically stated whether or not FP was allowed. However, both Christian and Muslim religious leaders referenced examples from religious texts and doctrine around fertility and family life that they interpreted to mean that FP use is acceptable. The texts referenced speak to principles around children, responsibilities of parents towards their children, and examples of practices described in religious texts.

Almost all Muslim religious leaders stated that FP for birth spacing was accepted in Islam and that they based this view on their interpretation of the Quran that says to breastfeed for two years, with some leaders calling it “required birth spacing” or viewing birth spacing to be “God’s intention.” Muslim religious leaders also mentioned that the example of withdrawal in hadiths and its use during the time of the Prophet was interpreted to mean it is approved by Islam. Some Muslim leaders further interpreted the approval of these methods to extend to the concept of FP and to modern methods more generally.

*“My point of view on the use of family planning*,* I think that planning or FP is a good thing because you must plan. It allows the mother to have good health*,* it also allows the child to have good health because even at the time of the Prophet (peace and blessings be upon him) where the modern methods that we have today did not exist they used coitus interruptus to be able to actually do this planning. So for me*,* from the moment Islam authorizes us to move in this direction it is a good thing*,* as an intellectual and a Muslim or a woman who has gone to school*,* it allows the woman to really plan to be able to follow her classes better*,* to be able to work better and manage her children.” -* Muslim religious leader from Ouagadougou (woman).

Second to religious doctrine, Christian and Muslim leaders discussed that their pro-FP stance was influenced by trainings on FP they attended and/or their own schooling and education. Religious leaders also spoke of the realities of modern life which include the financial and practical challenges of having and educating many children, the behavior of youth, and witnessing the suffering of women who do not space their pregnancies. A Christian leader spoke in detail about how FP use helps families avoid having more children than they can properly attend to. She listed the many needs children have in terms of health, nutrition, and schooling and how if these needs are not met, children are at risk of becoming street children. Lastly, some religious leaders mentioned how their own experience with birth spacing or being a part of a large family influenced their stance on FP. For example, a female Muslim leader who calls for flexibility in her religion to support FP use for birth spacing described her life circumstances where she had 24 siblings from her father’s multiple wives and the struggles her family faced in meeting the needs of all the children. She further went on to state that beyond her own experience, presently “life has become expensive” and “complicated” and thus women are more likely to need to use FP.

### Young women’s reasons for FP use

The young women were asked in the FGD about reasons why they might use FP despite their perception of their religion’s opposition towards FP. The reasons mentioned differed depending on the marital status of the group. For young married women, several reasons were mentioned that justified their FP use. The main reasons discussed were the difficulties inherent to modern life, mainly costs related to healthcare, food, and education for their children. Participants expressed that given the increasingly challenging economic context and living conditions, caring for children is becoming more complex and it is necessary to resort to FP use to space or limit births to take better care of their children. Having many children or children spaced closely together is a burden for women. FGD participants acknowledged that while they felt that their religion is not accepting of FP use, they said that their religious community will not come to help them if they follow their teachings and have as many children as possible.

*“They justify using it because people in the community will not come and help them raise their children. It’s true that you are part of a religion*,* but they will not come to help you. Even between you and your husband*,* it is the woman who suffers more with the child than the man.” -* Married Christian young woman from Ouagadougou.

*“…It is true that even if religion does not support it*,* we often have no choice*,* with living conditions which have become harsh*,* to have food we have to wake up very early to go sell and hope to have something to feed the family*,* at that moment you will have no choice but to space out your births*,* above all asking God for forgiveness.” -* Married Muslim young woman from Ouagadougou.

Married women also discussed using FP to preserve harmony in their household and marriage. Caring for many young children can place strain on a household and women may not be as available to take care of other household tasks or their husbands if they are preoccupied with childrearing. Some young women also mentioned fears that their husbands will be unfaithful or leave them if they are unhappy at home.

*“We have to use this*,* we have to plan births in order to be fulfilled. If you space your births*,* it gives you health*,* you enjoy your youth*,* you can take good care of your husband as they have already said. If you have births too close together*,* as soon as the husband comes home*,* a child has diarrhea and you have not yet finished changing him when the smell scares your husband away. Plus*,* it leads to a lot of arguments. Men love cleanliness*,* so we have to space out the children.” -* Married Christian young woman from Bobo-Dioulasso.

Unmarried young women discussed reasons for using FP despite their perception of their religion’s prohibition of premarital sex and FP for them; their reasons differed from those mentioned by married women. For unmarried young women, regardless of religion, the main reason for FP use was to avoid unintended pregnancy. Having a child outside of marriage can have significant social consequences for a woman and her family. FGD participants described young unmarried girls who become pregnant as bringing dishonor to their family. Unmarried young women who become pregnant or have children become victims of banishment and social exclusion and/or are looked down upon by their communities. To avoid this treatment for themselves and their families, young unmarried women choose to use FP to prevent unintended pregnancies.

*“What religion says about it [FP] can be an obstacle*,* it’s true that God says it’s not a good thing*,* but we do it hoping that God can forgive us; as a young woman if you don’t use FP and you get pregnant*,* your parents will kick you out of the yard*,* what will you do then? So you have to go and use contraception…”* Unmarried Muslim young woman from Bobo-Dioulasso.

*“Even if religion is against doing it*,* what can lead a young girl to do FP– if you are going out with a partner who cannot abstain. If he decides you have sex*,* you’re going to give in. If you too do not want there to be a child at the end of this sexual intercourse*,* you will necessarily do FP. So*,* it also depends on the partner*,* if he is not in favor of abstinence it will push you to adopt FP.” -* Unmarried Christian young woman from Bobo-Dioulasso.

Another reason unmarried young women use FP that was mentioned was to be able to continue their own studies. For young women who do not have support to help care for their child while in school, becoming a mother means they must end their schooling. Therefore, some young women choose to use FP to ensure they can complete their studies. A young married women described why in her opinion young unmarried women take recourse in FP:

*“Many hide their [FP] use*,* even the boys. If the person becomes pregnant*,* she will no longer be able to go to school*,* there will be no one at home to take care of her child. This is why they no longer ask someone’s opinion before using [FP]. They go to a health center to ask for advice on how to use it.” -* Married Christian young woman from Bobo-Dioulasso.

## Discussion

The results of this study show that young women of all faiths generally believe that their religion is against FP use. However, some young women did discuss a more open stance towards FP use for married women for the purposes of child spacing or the health and well-being of them or their child. Since the young women included in this study were FP users, they were asked about their reasons for using FP despite their perception that their religion does not allow it. Married young women choose to use FP due to the difficulties of modern life, the rising costs of child rearing, and to maintain harmony within their household. Unmarried young women choose to use FP to avoid social exclusion and consequences of an unintended pregnancy as well as to safeguard their own schooling.

IDIs with pro-FP religious leaders found that while interviewed leaders of all faiths were accepting of FP generally, they were emphatically against FP use for unmarried young people. However, in regard to their opinions about sharing information about FP, they expressed more diverse opinions with some leaders who felt it is fine to share information to prepare young women for their future married life and others who felt that providing information about FP should only be shared with married young people. Religious leaders expressed that FP use was acceptable for married women for the purposes of child spacing; this position was based on their interpretation of religious texts and teachings that they feel sanction FP. Religious leaders’ positions on FP were also influenced by their own life experiences.

The results of this study affirm much of what has been found in other studies on religion and FP acceptability and use. This study is of additional value as it examines the perspectives of both young women who currently use FP and religious leaders asked about perspectives on FP use in general and among young women. The data come from Burkina Faso, a francophone West African Country with a diverse mix of religions practiced; data come from the two largest cities—Ouagadougou (capital) and Bobo-Dioulasso. It is interesting to compare the perspectives of the young women and the religious leaders. In this case, the religious leaders acknowledge that FP use is acceptable for married young women, but this was not generally felt among the young women interviewed, married or unmarried. Generally, the young women did not report hearing positive messages from religious leaders and their teachings. This might explain the gap between young women’s perceptions and religious leaders’ actual beliefs. If more religious leaders are trained and share positive FP perspectives, at least the married young women who are using would not feel that they are going against their religion.

Another qualitative study with religious leaders of Catholic, Muslim, and Protestant faiths in Burkina Faso was conducted by Barro and Bado (2021) and found that religious leaders interviewed had knowledge of modern FP methods. Barro and Bado also found that religious leaders were supportive of birth spacing; however, they had a preference for natural and traditional methods of FP. Modern method use was considered to be for limiting births which was not permissible in their view [17]. The religious leaders in our study, while they spoke in greater depth about natural methods of FP including LAM, rhythm and withdrawal, they largely expressed an acceptance of modern method use, unlike what was found by Barro and Bado (2021). Furthermore, modern FP was not simply equated with the limitation of births and they considered it acceptable to use modern methods to achieve birth spacing.

The young women who participated in the focus group discussions did not perceive that their religion supported their FP use; however, they reported that factors other than religious approval were more influential in their decision to use FP. The need to space children due to increasing costs of raising and educating them along with the complexities of modern life and other benefits of child spacing for married women were more pressing than religious approval. For unmarried users, the desire to avoid unintended pregnancy and to prioritize their own schooling were the deciding factors in their contraceptive decision making. These findings echo the results of a study in Pakistan, where although Muslim men and women believed fertility control was in opposition to God’s will, most had tried to control their fertility due to reasons related to limited financial resources or to avoid the social stigma of having too many children [7]. The results of our study offer a different perspective from other studies such as a qualitative study in Tanzania that found that while there was some diversity in perceptions of religion’s acceptance of FP, religious approval of FP use was of central importance to the men and women interviewed [22]. The results of our study may denote that broader pressures at the societal level exert a stronger influence on young women’s decisions around FP use than religious doctrine does in urban Burkina Faso.

Our findings highlight the fact that young women have need for and are using FP, despite their perceptions of religious opposition. While religious leaders oppose sex and FP use before marriage, adolescents and young women are engaging in these behaviors. Religious leaders’ expectation that unmarried young people practice abstinence may no longer be practical or realistic. An analysis of Demographic and Health Survey (DHS) data from 31 Sub-Saharan African countries showed that 21.3% of adolescents aged 15–19 had premarital sex in the past year. Furthermore, the DHS analysis showed that adolescents and young women are getting pregnant with 29.2% of women 20–24 having initiated childbearing before age 18, and among women under 20, 25.5% of births were unintended [25]. Religious leaders in our study expressed reservations about discussing and educating unmarried women about FP methods, but young and unmarried women have sexual and reproductive needs that require access to FP information and services.

Religious leaders can play an important role in reducing barriers to FP services and care. Through the lens of the SEM, religious leaders act at the community level by influencing norms and expectations within religious communities. Furthermore, as McQuillan outlines as the second mechanism through which religious leaders are influential, they have a platform within the community through which they can communicate these norms. For example, in a study among urban Nigerian women, it was found that those who were exposed to favorable FP messages from religious leaders were significantly more likely to adopt a modern method [26]. 

This study has several limitations. First, the study uses qualitative data that cannot be generalized beyond the study participants and sites. Second, the religious leaders who were selected to participate in the study were “pro-FP” and their points of view may not reflect the opinions of other religious leaders who may be less open to FP. Lastly, the young women who participated in the study were all FP users; therefore, the study does not consider insights from non-users who may be more affected by religious leaders or influenced more strongly by their religious community.

## Conclusion

While young Christian and Muslim women from the Burkina Faso sites of study largely perceive their religion to be against FP use, nuance exists in religious leaders’ positions and their interpretations of their religion’s stance on FP. It is important for religious leaders to understand that young women both married and unmarried are using FP, even if they do not perceive it to be permitted religiously. Religious leaders and other stakeholders can consider sharing messages on the acceptability of FP use to bring into alignment these leaders’ evolving beliefs about FP and young women’s contraceptive decision making and needs.

## Supplementary Information

Supplementary Material 1. IDI guide_religious leaders; Word file; In-depth interview guide for religious leaders

Supplementary Material 2. FGD guide_young women; Word file; Focus group discussion guide for young women

## Data Availability

The datasets generated and/or analysed during the current study are not publicly available in order to protect the identities of the participants but are available from the senior author (speizer@email.unc.edu) on reasonable request.

## Abbreviations

FGD: Focus group discussion
FP: Family planning
IDI: In-depth interview
ISSP: Institut Supérieur des Sciences de la Population
LAM: Lactational amenorrhea method
SEM: Social-ecological model
UNC-CPC: Carolina Population Center at the University of North Carolina, Chapel Hill
URCB: Union des Religieux et Coutumiers du Burkina
