# Supplementary material for: Perspectives of religious beliefs and family planning by religious leaders and young women: results from a qualitative study of Bobo-Dioulasso and Ouagadougou in Burkina Faso
Source: BMC Public Health. 2026 Jan 8;26:488. doi: 10.1186/s12889-025-26160-z (PMC12882276; doi:10.1186/s12889-025-26160-z)
Supplement: Supplementary file 1 — Supplementary Material 1. IDI guide_religious leaders; Word file; In-depth interview guide for religious leaders [file 12889_2025_26160_MOESM1_ESM.docx]

**Role of religion and religious leaders on contraceptive use among adolescents and youth**

**IN-DEPTH INTERVIEW GUIDE**

**Religious leaders (Male/female according to denominations)**

**Introduction (Presentation of the objectives and purpose of the study; presentation of expectations of the respondent)**

*[Researcher] Once again, thank you for agreeing to participate in this discussion today. I'm going to ask you some questions related to the role of religion and religious leaders on contraceptive use among adolescents and young people. Keep in mind that if you don't want to answer some of the questions I'm going to ask you, you are free to skip them.*

Introduction:

1. Please tell me about the role(s) you play within your faith community.
   1. How long have you been working within your faith community? How have these roles changed over this time?
2. Please share with me your experiences working with youth within your faith community.
   1. How have your experiences working with youth changed over time?

Personal experiences:

1. As a religious leader within your faith community, what are your views on family planning use?
   1. What was your journey to these current views on family planning use?
   2. Who or what influenced your views on FP?
2. As a religious leader within your faith community, what are your views on young people (aged 15-24) being provided with sexual and reproductive health information and services, including family planning?
3. Probe: if family planning not specifically discussed: what are your opinions about young people using family planning?
4. What was your journey to these current views on family planning use among young people?
5. Who or what influenced your views on FP?
6. How do your views on young people differ based on sex?
7. What are your views on young men (aged 15-24) using family planning? How about for young women? Why do you feel this way (i.e., same or different)?
8. How do your views on young people using family planning change with marital status? For those unmarried youth? Married youth?
9. Does sex play a role in your opinions based on marital status (e.g., unmarried young women vs. married young women and unmarried young men vs. married young men)?
10. How does parity influence your views on youth family planning?
11. Are there specific contraceptive methods that are more favorable for youth to use within your religious views? Less favorable? Why?
    1. Does the more favorable method for young people differ by marital status and parity?
12. Have you ever been involved in activities to promote or encourage the use of FP among adolescents and young people? If so, please describe to me your participation in these activities.
    1. Have you ever been involved in activities to discourage the use of FP among adolescents and youth? If so, please describe what you were campaigning against, the messages, activities and your participation in those activities.
13. In what settings do you talk about FP?
14. With your greater congregation? Smaller groups? Youth groups?
15. Are there some topics that you reserve for specific settings?
16. Do you ever speak with parents or the wider community about young people’s contraceptive use?
    1. How do you speak with parents about their youth’s contraceptive use?
    2. Please share an example of a time when you spoke with a parent about their concerns for their youth (aged 15-24) who was using or thinking of using family planning. What was successful about that interaction? What was not?
17. How do you think young people view their faith community in terms of their access to and use of contraception? Do they see it as a barrier, a facilitator, or is not relevant to their decision-making?
    1. Do you have ideas of ways to address this through your teachings and engagement with young people?

Religion and Contraceptive Use:

1. In your opinion, are there any passages in the Bible or the Koran that would be favorable to the use of FP by women? By young people? Which? If not, why?
2. Are there parts of your religious texts that you cite while advising youth on contraceptive use?
3. Do your religious schools (Koranic school, Catholic school, etc.) have components on sex education? How do they approach teaching youth about family planning?

Consultation between religious leaders on FP issues:

1. How do you work with other religious leaders to promote FP for adolescents and youth in your community?
2. What has been successful about your engagement with other religious leaders? Challenging? Why?
3. Are there discussions within your religious associations about how to approach sex, sexuality, and FP among young people? If so, would you please describe to me these discussions.
4. What disagreements are there? On what issues do people agree?
5. Among your peers that do not believe in youth contraceptive use, why do you think they feel that way?
6. Please share with me an example of an interaction with a religious peer who did not share the same opinion as you about youth family planning.

Difficulties faced by religious leaders in promoting AY FP use:

1. What are the barriers that you have faced as a religious leader promoting FP for AY?
2. Have you ever felt pressured to be against AY FP use? If yes, please share with me an example of when this happened and how you reacted to it.

Strengthening support for religious leader’s support for FP:

1. Did you receive training or support for your work on family planning in the community and with young people? Where did this support come from and how do you engage with these partners? Did you find this training/support helpful or not?
2. What support/education/mentorship do religious leaders need to become FP champions for their youth congregation?
3. How do you think religious leaders and others in the faith community could promote youth attendance and better access to FP services?

Conclusion:

1. What have we missed today in our discussion about youth family planning and religion that would be important for us to know?
2. What questions do you have for us?
