# Supplementary material for: Perspectives of religious beliefs and family planning by religious leaders and young women: results from a qualitative study of Bobo-Dioulasso and Ouagadougou in Burkina Faso
Source: BMC Public Health. 2026 Jan 8;26:488. doi: 10.1186/s12889-025-26160-z (PMC12882276; doi:10.1186/s12889-025-26160-z)
Supplement: Supplementary file 2 — Supplementary Material 2. FGD guide_young women; Word file; Focus group discussion guide for young women [file 12889_2025_26160_MOESM2_ESM.docx]

**Role of religion and religious leaders on contraceptive use among adolescents and youth**

**Young women focus group discussions**

**Introduction to Focus Group Participants**

Welcome. Thank you for being here. My name is ____________ and I am part of a research team working with _________. As we explained during the informed consent process, we are trying to understand adolescents’ and young women’s perspectives and experiences related to the role of religion and religious leaders on contraceptive use. The information you provide will be used to develop or improve health programs for adolescent girls and young women. We will not use your names or otherwise identify you personally in the analysis and reporting of the results of our discussion today.

**Ground Rules**

We encourage everyone to participate, knowing there are no right or wrong answers. Speak up if you agree or disagree with someone, as we want to understand everyone’s opinions. Everyone in this room has agreed to keep the conversation confidential and not share any information with others after the session is over. Please do not share any personal details; we will assign each of you a number so you do not need to use names. We will record this discussion to help us capture everything we need for the analysis and summary, but we will not identify you personally. This discussion will probably take about an hour and a half. Do you have any questions before we get started?

**Warm Up**

1. We would like to hear from you about what role religion plays in influencing your daily lives including affecting where you go, who you spend time with, and what you do with your free time?

**FP in the community**

1. How is family planning normally discussed within your faith community?
   1. Where do these discussions typically take place?
   2. When do these discussions typically take place?
2. Are there people in your religious community to whom young women can go to for family planning advice?
3. Who are these people and what makes them useful advisors on family planning?
4. What do you think your religion's position is on family planning?
5. Are there different opinions within your religion on how people view family planning? What are the different opinions?
6. How is your religion’s view on family planning different now than when your parents were your age?
7. How does your religion serve as a barrier to young women who want to use family planning?
8. How do young religious women overcome these barriers?
9. What factors serve as justifications or rationale for deciding to use FP if young women believe their religion is not supportive of FP?
10. When is your religion supportive of young women’s use of family planning?
11. What does your religious community say about family planning use by **unmarried** young women?
12. Does your religious community feel differently if the unmarried woman has already had a child? How is it different?
13. What does your religious community say about family planning use by **married** young women?
    1. Does your religious community feel differently if the married woman has already had a child? How is it different?
14. Are there certain types of family planning methods that are more acceptable by your religion for young women to use? Why?
15. Are there certain methods that your religion feels are appropriate for **unmarried** young women? Does it matter if she has a child already?
16. Are there certain methods that your religion feels are appropriate for **married** young women? Does it matter if she has a child already?
17. What are the perceived consequences within your religious community to a young woman of your faith for using family planning? Probe for positive consequences and negative consequences.
18. Does it matter if she is unmarried or does not have children?
19. What would be more unfavourable in your religious community: a young, unmarried woman who gets pregnant or the same young unmarried women who uses family planning? Why?
20. Do traditional religious beliefs influence young people’s decision-making about family planning use?
    1. How do young people balance their traditional beliefs with their other religious beliefs?
    2. Is formal religion or traditional religious beliefs more influential on young women’s family planning decision-making?
    3. Is formal religion or traditional religious beliefs more influential on a young person’s choice of what method to use?
21. How can we make it acceptable for all young women (married or unmarried) in your religious community to seek contraceptive counselling and use family planning when or if they need it?

Conclusion:

1. What have we missed today in our discussion about youth family planning and religion that would be important for us to know?
2. What questions do you have for us?
